# Rosuvastatin calcium nanoparticles: Improving bioavailability by formulation and stabilization codesign

**DOI:** 10.1371/journal.pone.0200218

**Published:** 2018-07-09

**Authors:** Doaa H. Alshora, Mohamed A. Ibrahim, Ehab Elzayat, Osaid T. Almeanazel, Fars Alanazi

**Affiliations:** 1 Kayyali chair for Pharmaceutical Industries, Department of Pharmaceutics, College of Pharmacy, King Saud University, Riyadh, Saudi Arabia; 2 Department of Pharmaceutics, College of pharmacy, Al-Azhar University, Assiut, Egypt; VIT University, INDIA

## Abstract

**Purpose:**

Rosuvastatin calcium (ROSCa) is a poorly soluble drug with bioavailability not exceeding 20%. Decreasing the particle size may enhance its solubility, dissolution rate and bioavailability. Therefore, the aim of the current study is to prepare ROSCa nanoparticles by wet milling technique using planetary ball mill. The codesign between formulation and stabilization of nanoparticles was studied to achieve both high dissolution as well as bioavailability.

**Methodology:**

ROSCa nanosuspensions was prepared by wet milling technique using planetary ball mill, by applying milling ball size of 0.1 mm at speed of 800 rpm for 3 cycles each cycle composed of 10 minutes. HPMC, PVP k-30, pluronic F-127, Tween 80 and PEG 6000 were used as stabilizers. The nanosuspensions were then freeze-dried, and the dried nanoparticles were evaluated for particle size, zeta potential, *in-vitro* dissolution test, XRPD and *in-vivo* study.

**Results:**

ROSCa nanoparticles stabilized with 10% PVP (P3) had a good stability with smallest particle size, which in turn enhanced the dissolution rate. The particle size of the leading formula was 461.8 ± 16.68 nm with zeta potential of -31.8 ± 7.22 mV compared to untreated drug that has a particle size of 618μm. The percent of ROSCa dissolved after 1 hour enhanced significantly which reached 72% and 58.25% for leading nanoparticle formula and untreated ROSCa, respectively (*P* < 0.05). The *in-vivo* study of ROSCa from the leading nanoparticle formula showed a significant enhancement in the C_max_ after 2 h (82.35 ng/ml) compared to 9.2 ng/ml for untreated drug.

**Conclusion:**

Wet milling technique is a successful method to prepare ROSCa nanoparticles. From different stabilizer used, PVP (10%) was able to produce stable nanoparticle with small particle size which significantly enhance the dissolution rate and pharmacokinetics parameters of ROSCa.

## 1. Introduction

Rosuvastatin calcium (ROSCa) is the most effective antilipidemic drug and is called "super-statin". It decreases the low-density lipoprotein-C (LDL-C) by 63% after an administration of 40 mg dose [1, 2]. ROSCa is a poorly water-soluble drug with only 20% oral bioavailability. It is classified by biopharmaceutical classification system as class II drug. The poor solubility of ROSCa affects its dissolution rate and, in turn, its bioavailability. Thus, enhancing the dissolution of ROSCa can lead to improve its oral bioavailability. Accordingly, several nanosization approaches were adopted to enhance ROSCa dissolution and bioavailability.

The principle of nanosization technology is based on size reduction. Drug nanoparticles are drugs in a nanosize range [3]. Krishnamoorthy et al. [4] prepared self-nanoemulsifying delivery system of ROSCa that showed improved solubility and oral bioavailability by overcoming the hepatic first-pass metabolism. Gabr et al. [5] investigated the elaboration of ROSCa loaded nanosponges for enhance its oral bioavailability. ROSCa-loaded NS were prepared by lyophilization technique by using a molar ratio of 1:6 of β-CD: pyromellitic dianhydride. Results of nanosponges indicated highest entrapment efficiency, an optimum particle size, a narrow size distribution, and higher zeta potential leading to good colloidal stability with enhanced dissolution rate as well as improved bioavailability. Additionally, size reduction to nano-size range was also reported for ROSCa by using high pressure homogenization (HPH) and precipitation technique [6]. The results showed enhancement of dissolution by 36% and bioavailability by 1.87-fold. Noticeably, the smallest obtained particle sizes of the ROSCa were 749 nm and 2000 nm for HPH and precipitation technique, respectively. Therefore, applying other nanosization techniques, to reach smaller size having more dissolution rate and bioavailability, is needed.

The milling has been reported as a highly effective technique. It is a process of applying mechanical energy that affects particle size and thus specific surface area as well as shape of drug particles [7]. Prolonged transfer of mechanical energy to drug particles provides mechanochemical activation leading to disordering of the crystal structure and amorphization [8]. Milling by using planetary ball mill has been used recently to enhance the dissolution rate and bioavailability of poorly-water soluble drugs [9, 10].

The aim of production of nanosuspension is to enhance both dissolution rate and bioavailability of ROSCa. However, physical stability of the prepared nanosuspension during preparation and shelf life storage should be ensured. The major problems of nanosuspension instability can be simplified as the aggregation of small particle size after size reduction. This is also known as Ostwald ripening in which small particles are recrystallized and forming large particles.

The addition of stabilizers can enhance the physical stability of the nanosuspension by different mechanisms according to its properties. Ionic surfactant stabilizers stabilize the colloidal system by electrostatic effect [11]. The ionic stabilizers adsorb on the particle surface leading to formation of repulsive forces between the particles [12]. Differently, stabilization of the colloidal system also can be done by using polymers and non-ionic surfactants exerting a steric effect. Most of the polymers contain hydrophobic and hydrophilic moieties in their structures such as HPMC, PVP and Ploxamer. The hydrophobic moiety adsorbs on the particle surface, while the hydrophilic one remains in the dispersion medium [13]. Combination of both electrostatic and steric stabilizers also could be used exerting an effect called electrosteric stabilization.

The aim of the present study was to prepare and characterize ROSCa nanoparticle by planetary ball milling technology to enhance both its dissolution rate as well as bioavailability. The effects of type and concentration of the nanoparticles stabilizers were also investigated. Design jointly (codesign) of enhancing the stability and dissolution by stabilizers was targeted in this study.

## 2. Materials and methods

### 2.1. Materials

Rosuvastatin calcium (ROSCa) was purchased from Beijing Mesochem Technology CO. Ltd. (Beijing, China). Hydroxypropyl methylcellulose, HPMC (Methocel E5 PREM LV) was obtained from DOW (Midland, MI, United States). Polyvinylpyrrolidone (PVP K30, M.Wt. 40000) was obtained by Loba Chemie (India). Tween^®^ 80 was purchased from Merck Company (Muenchen, Germany). Poloxamer 407 (Pluronic F127^®^) was obtained from C.H. Erbesloh (Krefeld, Germany). Other chemicals were of reagent grade and were used as received.

### 2.2. Methods

#### Preparation of ROSCa nanosuspensions

Prior to wet milling, ROSCa was subjected first to dry milling using planetary ball mill (Pulverisette 7 Premium, Fritsch Co. Germany). This step was done because the particle size of the starting material subjected to wet milling by the planetary ball mill was 618 μm. Zirconium balls with a size of 5 mm were used as the milling medium for the dry milling procedures in powder: ball ratio (1:10). The dry milled drug was further nanosized by wet milling.

For the wet milling procedures, definite weight of dry milled ROSCa was mixed with the milling solution; water (with or without stabilizer) in different ratios. The dispersion was then nanosized by using zirconium ball of 0.1 mm size and milling speed of 800 rpm. This also done using three milling cycles (10 min of each) with a pause of 5 min after each milling cycle. The effect of stabilizers types namely: HPMC, PVP, Tween 80, Pluronic F-127 and PEG 6000 as well as their concentrations on the produced nanoparticles was also investigated. The prepared nanosuspension was subjected to freeze- drying and then solid nanoparticles was stored in a tight container at -30° for further characterization.

#### Drug content

In 10 ml volumetric flask, 10 mg of nanoparticles was dissolved in 10 ml methanol. One ml was taken in 10 ml volumetric flask and completed to the volume with 0.1N HCl. The absorbance was measures at λ 244 nm against a blank made of stabilizer only following the same procedure. The drug content experiment was carried out in triplicate.

#### Particle size and zeta potential

The particle size and zeta potential **ζ** of the nanosuspension were determined by using Malvern zetasizer (Nano S, Worcestershire, UK).

#### X-ray powder diffraction (XRPD)

The crystallinity of the pure drug and nanoparticles was determined by using X-Ray diffractometry. The X-ray diffraction patterns of the powder samples were obtained using RIGAKU diffractometer (Tokyo, Japan) which is equipped with curved graphite crystal monochromater, automatic divergence slit and automatic controller PW/1710. The target used was CuKα radiation operating at 40 KV and 40 mA (λkα = 1.5418 Å). The diffraction pattern was achieved using continuous scan mode with 2Ɵ° ranging from 4° to 60° [14].

#### Fourier transform infrared spectroscopy (FTIR)

Fourier transform IR spectra were recorded for ROSCa raw material and nanoparticles. The samples were grounded separately and mixed thoroughly with potassium bromide. The ratio of sample and KBr was kept for all the preparations. The potassium bromide discs were prepared by compressing the powders at a pressure of 5 tones for 5 min in a hydraulic press. Scans were obtained from 4000 to 500 cm^−1^.

#### *In vitro* dissolution study

The *in-vitro* dissolution profile of ROSCa nanoparticles was studied using a USP-II dissolution apparatus (Pharma Test, DT 70, Germany). The dissolution experiment was carried out in 500 ml of 0.1 N HCl as a dissolution medium at 37 C° and 50 rpm. Five ml aliquot samples were withdrawn at pre-determined time intervals (5, 10, 15, 30, 45 and 60 minutes) using poroplast-kerze filter and diluted suitably. The absorbance was measured spectrophotometrically at 244 nm against a suitable blank.

Dissolution efficiency after 60 min (%DE_60_) was measured using the trapezoidal rule calculated from the area under the dissolution curve at time t and expressed as percentage of the area of the rectangle described by 100% dissolution in the same time [15]. Also, the relative dissolution rate (RDR_60_) of the dissolution was calculated by determining the amount of ROSCa dissolved from tested samples and normalizing for the amount of drug dissolved from pure drug sample over the same time (60 minutes).

#### Pharmacokinetic studies

***In-vivo* and pharmacokinetics analysis of ROSCa nanoparticles**:

Animal: Rabbits (weight 2.5–3 kg) were obtained from the College of Pharmacy experimental animal care center (King Saud University, Riyadh, Saudi Arabia). The animals were fasted for 24 hours prior the administration of the drug, the drug was administered using the oral route by oral gavage in order to assure the dose uniformity. The study was approved by the Ethical Committee (Protocol no. 80–23) by Experimental Animal Care Unit. The experimental protocol was in agreement with the Guide of the National Institution of Health (NIH) for the Care and Use of Laboratory Animals.

Drug administration: Two groups of animals were administered the calculated animal dose equivalent to 10 mg. Both raw ROSCa and nanoparticle formulation were suspended in 0.5% Na-CMC just before administration and administered orally via gastric tube. Aliquots of blood sample were collected at different time intervals for at least 24 hours. The blood samples were treated and analyzed using developed UPLC-MS/MS method [16]. Different pharmacokinetics parameters were calculated such as C_max_, T_max_, AUC, rate of absorption and rate of elimination using a non-compartmental model analysis.

Blood sampling: At specific time interval (1, 2, 3, 4, 6, 8, 12 and 24 h), 1 ml blood was withdrawn from ear vein in heparinized tube. The blood sample was centrifuged at 6000 for 20 minutes to separate the plasma.

**Analysis of ROSCa in rabbit plasma**:

Sample preparation: The plasma samples were processed by protein precipitation method. Simply, 0.2 ml of plasma sample was combined with 50 μl internal standard Prednisolone (100 μg/mL) and 0.75 ml methanol, then vortexed for 1 min. After centrifugation at 15,000 rpm (15,000×g) for 10 min, 750 μl of the supernatant liquid was transferred to a sample vial and 5 μl was injected into the LC/MS/MS for quantitative analysis. Chromatographic conditions: In this study, a validated UPLC-MS/MS (UPLC: Waters Acquity, Milford, MA, USA) was employed to determine the concentration of Rosuvastatin in rabbit plasma [16]. The chromatographic conditions involved the use of a BEH C18 column (50 mm x 2.1 mm, 1.7 μm) with a mobile phase of acetonitrile and 0.1% formic acid (35:65, volume: volume) at a flow rate of 0.25 ml/min using prednisolone as the internal standard. The eluted compounds were detected by tandem mass spectrometry using TQ detector (Waters Corp., Milford, MA) equipped with an electrospray ionization (ESI) source operating in positive ionization mode. The quantification was performed with multiple reactions monitoring (MRM) mode. Selection of ionization pairs (m/z) was shown as follows: Rosuvastatin: 482.097→258.072 (cone voltage 60 V, collision energy 34 V), Prednisolone: 403.172→385.224 (cone voltage 42 V, collision energy 13 V).

#### Statistical analysis

One way analysis of variance (ANOVA) was used for comparison. A value of p < 0.05 was denoted significant throughout the current study.

## Results and discussion

### Impact of stabilizer type and concentration on particle size

**Fig 1:**
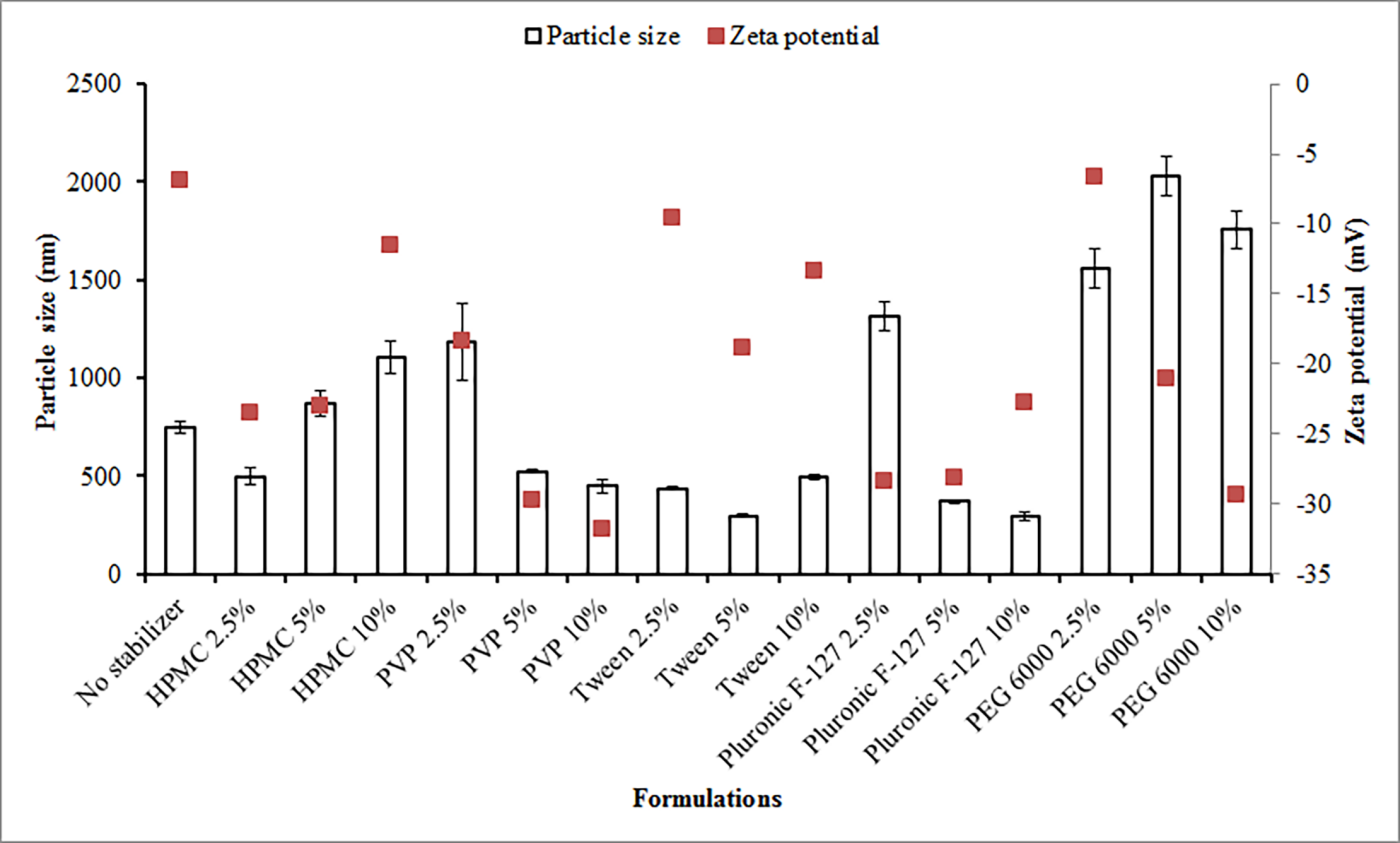
Effect of different types and concentrations of stabilizers on the particle size and zeta potential of ROSCa nanosuspensions prepared by planetary ball mill, compared to the untreated ROSCa powder (618 μm).

The effect of different stabilizers (HPMC, PVP, Tween 80, Pluronic F-127, PEG 6000) and their three level of concentrations (2.5%, 5%and 10%) on the particle size of ROSCa nanosuspensions were studied (Fig 1). Untreated ROSCa nanosuspension showed particle size of 618 μm. The particle size of ROSCa nanoparticles prepared by planetary ball milling without using stabilizer is 750 nm. It was noticed that the produced nanosuspensions in absence of a stabilizer are prone to be increase in size due to the high surface free energy on their surfaces that might result in particle aggregation [17]. Therefore, the use of stabilizer in the preparation of nanosuspension is required.

The particle size of nanosuspension stabilized with 2.5% HPMC was 500 ± 45.31 nm, which is lower than particle size observed in case of non-stabilized nanosuspension (750 ± 20.66 nm). Increasing HPMC level in the nanosuspension from 2.5% to 5% and 10% resulted in enlarging particle size to be 869.2 ± 63.99 nm and 1105 ± 81.79 nm, respectively. Increasing HPMC concentration might result in increasing solution viscosity, retardation in the movement of milling balls, and resulting in larger particle size [18]. The enlargement of the particle size with increasing the HPMC concentration could also be due to the insufficient amount of polymer available to cover new large surface of particles due to increased viscosity and at this point the agglomeration can take place [19].

Fig 1 shows that increasing the concentration of PVP added to the formula decreased the nanosuspension particle size. Nanosuspension formulation with low PVP concentration (2.5%) had a larger particle size (1184 ± 198.69 nm), this particle size decreased with increasing the concentration of PVP to reach 448.3 ± 31.6 nm upon using 10% PVP. Similar findings were obtained by Nijlen and coworkers [20], who found that the particle size of artemisinin decreased with increasing the concentration of PVP from 50 to 67%. PVP stabilizes the nanosuspension by steric stabilization, in which the hydrophobic part of the polymer is adsorbed onto the particle surface. This led to cover the particle and prevent particles aggregation [21]. By increasing the PVP concentration in the medium, a well and firm structure was formed on the particle surface. At the same time, the hydrophilic chain in the bulk medium helps keeping the particle away from each other. Therefore, PVP showed a good stabilization property for the system by being adsorbed on the particle surface and inhibiting the crystal growth [22].

The three concentrations used of tween 80 as suspension stabilizers decreased the particle size of ROSCa nanosuspensions to range of 500 nm. Interestingly, the optimum surfactant concentration in decreasing the particle size was the 5% w/v, in which the particle size of 296.4 ± 9.62 nm was obtained.

The low concentration of the tri-block co-polymer; pluronic F-127 (2.5%) did not produce a nanosized particles (1316.33 ± 73.2 nm). Differently, the higher concentrations (5 and 10%) were significantly produced nanoparticles (370 ± 8.7 nm and 297.66 ± 22.64 nm, respectively). Pluronic F-127 at higher concentrations was able to decrease the aggregation of the particles by adsorption on the particle surface. The hydrophobic polypropylene oxide moiety may be adsorbed on the particle surface. In addition, the hydrophilic portion provides a steric hindrance property which is preventing the aggregation of the particles [23].

All concentrations of PEG 6000 did not result in decreasing the particle size to nanosize range. This could be due to the high hydrophilicity of the polymer which prevents the adsorption on the particle surface for steric stabilization.

### Zeta potential of ROSCa nanosuspensions

Zeta potential (**ζ**) can be defined as the potential difference between the stationary layer of fluid attached to the particles and the dispersion medium [24]. The stability of the colloidal system could be affected by the particle charge which is measured by the electrophoretic mobility of the particles in an electrical field (zeta potential). The charge on the particle surface could be occurred due to the dissociation of some functional groups which are mainly depending on the pH of the suspension. Zeta potential value between (-20 and -30 mV) indicates stable suspensions [25]. The zeta potential of ROSCa nanosuspension not containing stabilizer was measured and was found (-6.91 mv), (Fig 1). This low value of zeta potential indicates that this nanosuspension system is thermodynamically unstable and the addition of stabilizer is a must in order to increase its physical stability.

The effect of different stabilizers on zeta potential was investigated (Fig 1). The results showed that ROSCa nanosuspensions stabilized with different concentrations of PVP had a higher absolute zeta potential values than that of other stabilizers. The value of zeta potential for PVP stabilized nanosuspension became closer to (-30 mV) with increasing the PVP concentration. Nanosupension stabilized with 5% PVP had a value of (-29.7 ± 6.52 mV). Similarly, by increasing the concentration to10%, the absolute value of zeta potential was observed (-31.8 ± 7.22 mV), indicating the stability of the system.

The absolute value of zeta potential of nanouspensions stabilzed by pluronic F127 decreased with increasing the polymer concentration. Moreover, The zeta potential value for ROSCa nanosuspensions stabilized with different concentrations of HPMC were lower than that of stabilized with PVP and Pluronic F-127. The zeta potential values for nanosuspension formulations containing 2.5% and 5% HPMC were almost the same (-23.5 ± 4.96 and -23.0 ± 7.37 mV, respectively). This value decreased to (-11.5 ± 5.77 mV) with increasing the HPMC concentration to 10%. Additionally, the zeta potential of nanosuspensions stabilzed by PEG 6000 increased from (-6.6 ± 4.81 mV) to (-29.4 ± 7.51 mV) by increasing the stabilzer concentration in the nanosuspension from 2.5% to 10%, respectively.

Tween 80 stabilized nanosuspensions showed the lowest zeta potential values compared with other stabilizers. The low value of zeta potential value of these nanosuspensions (< -20 mV) could be due to the formation of thick adsorbed layer. This is due to the adsorption of the non-ionic surfactant to the nanosized particles resulting in covering and masking of the particle charge [26].

### Effect of freeze-drying

**Fig 2:**
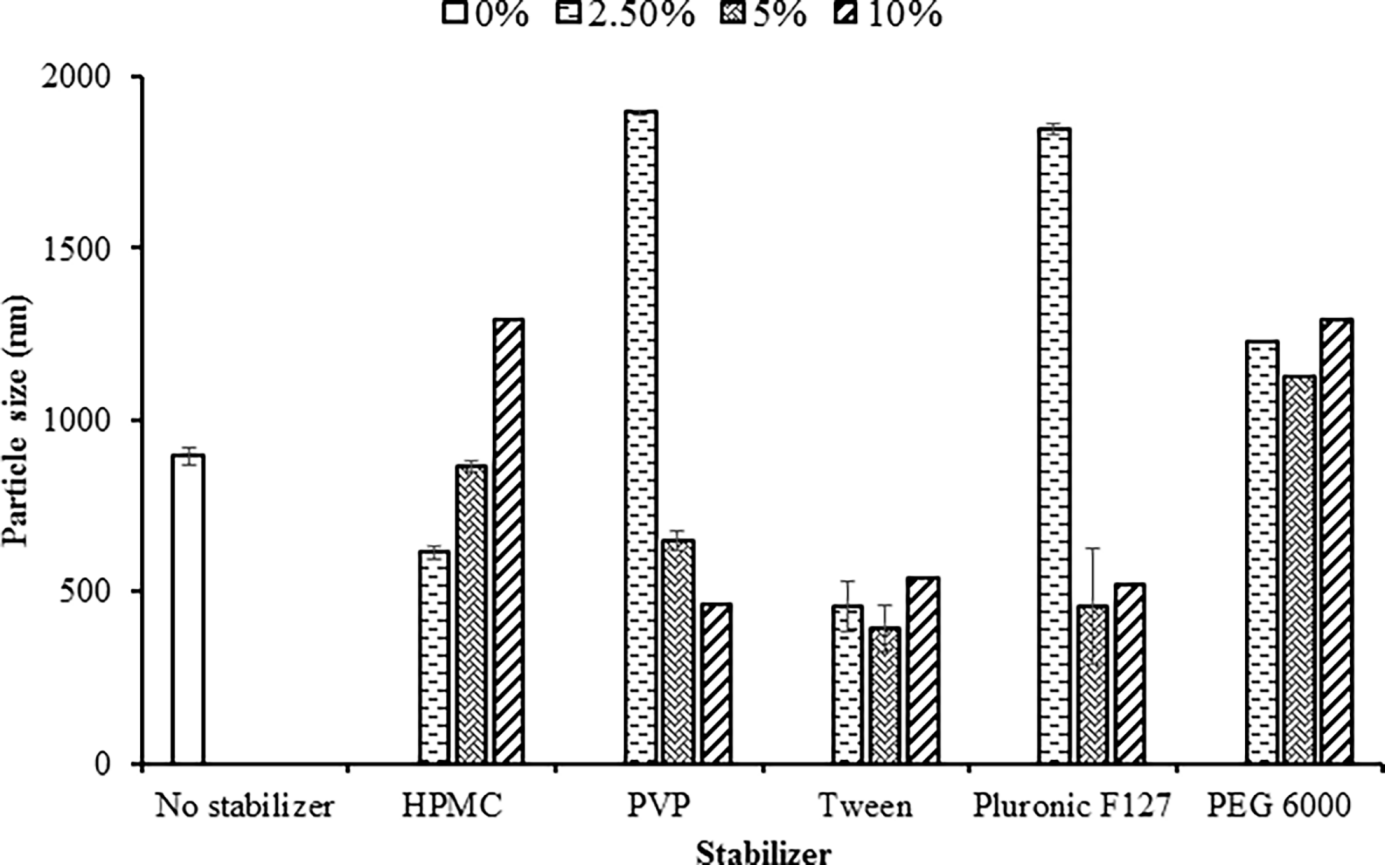
Effect of different types and concentrations of stabilizer on the particle size of the freeze-dried ROSCa nanoparticles prepared by planetary ball mill compared with untreated ROSCa (618 μm).

Fig 2 shows that the effect of freeze-drying on the nanoparticles stabilized by different polymeric stabilizers. Nanosuspensions stabilized by HPMC using lower concentrations (2.5% and 5%) exhibited slight change in size (499 to 611 nm and from 869 to 850 nm). Also, it was noticed that freeze-drying of nanosuspensions stabilized by 10% HPMC concentration resulted in enlarging particle size (1105 to 1289 nm).

Differently, freeze-drying of nanosuspension containing low concentration of PVP produced enlarged particles 1895.5±70 nm, while nanosuspensions stabilized with 5% and 10% PVP showed slight increase in their sizes upon freeze-drying. This could be attributed to high polymer weight ratio, which inhibits or at least decreases the mobility of drug particles and thus, inhibits its recrystallization [27]. Moreover, the size of freeze-dried nanoparticle formulations stabilized by 2.5% pluronic F-127 was found larger than the corresponding nanosuspension. However, no noticeable changes could be observed for nanoparticle formulations stabilized by the higher polymer ratios upon freeze-drying. This could indicate that ROSCa stabilized with 2.5% pluronic F-127 can’t withstand the lyophilization condition and it may cause a stability problem. The enlargement of the freeze-dried particle size could be attributed to strong inter and intra molecular hydrogen bonding which may lead to particle aggregation after reconstitution [28]. Similarly, freeze-drying of nanosuspensions stabilized by all Tween 80 concentrations did result in obvious changes in particles sizes. It was also found that freeze-drying of nanosuspension resulted in a fluffier powder with smaller particle size as such observed with ROSCa nanoparticles stabilized with PEG 6000.

### Non-milled suspensions in different polymeric solutions

**Fig 3:**
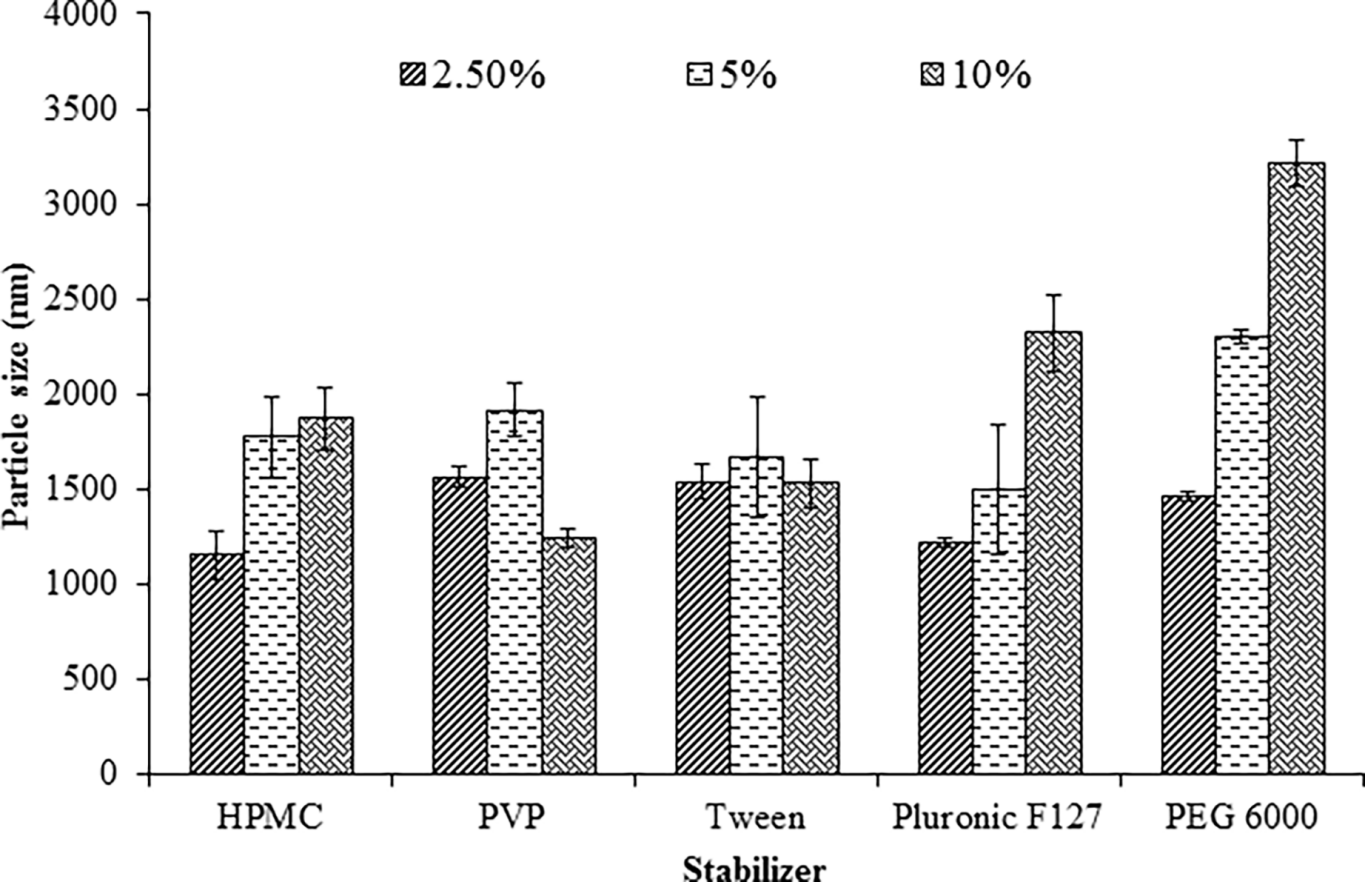
Effect of different types and concentrations of polymeric solutions on the particle size of ROSCa non-milled freeze-dried suspensions compared with untreated ROSCa (618 μm).

Freeze-dried suspensions containing drug and different concentrations of stabilizers were prepared without milling. This step was done in order to investigate if the reduction in the particle size is due to the milling process or the properties of the polymer. The effect of stabilizer type and concentration are presented in Fig 3. The used stabilizers were able to decrease the particle size of ROSCa in comparison to that of untreated ROSCa particle size (618 μm). Obviously, the reduction in the particle size did not produce particles in the nanosize range as the planetary ball milling procedures did (Fig 1). Suspending the drug in a solution of lower HPMC concentration (2.5%) without milling resulted in a decrease of the particle size to 1154.4 ± 127.9 nm. Further increase in HPMC level did not result in decrease in the particle size. On the other hand, ROSCa non-milled suspension in different PVP solutions (2.5%, 5% and 10%) exhibited a pronounced reduction of the drug particle size compared to the raw material after freeze-drying. ROSCa showed particle size of 1.5 ± 56.30 μm, 1.9 ± 136.47 μm and 1.2 ± 54.45 μm when it was suspended in 2.5%, 5% and 10% solution of PVP, respectively. Also, non-milled suspension containing 2.5% pluronic F-127 produced particle size of 1.214 ± 24.0 μm compared to 2.322 ± 202 μm for non-milled suspension that contains 10% pluronic F-127. Moreover, non-milled suspension containing PEG 6000 with different concentrations showed that as other tested stabilizers, the polymer decreased the particle size noticeably, but it produced the largest particle size comparing with other stabilizers [29]. Therefore, the effect of milling process on the drug suspension in different stabilizer solutions was attributed to decrease the drug particle size to nanoscale.

### *In-vitro* dissolution

**Fig 4:**
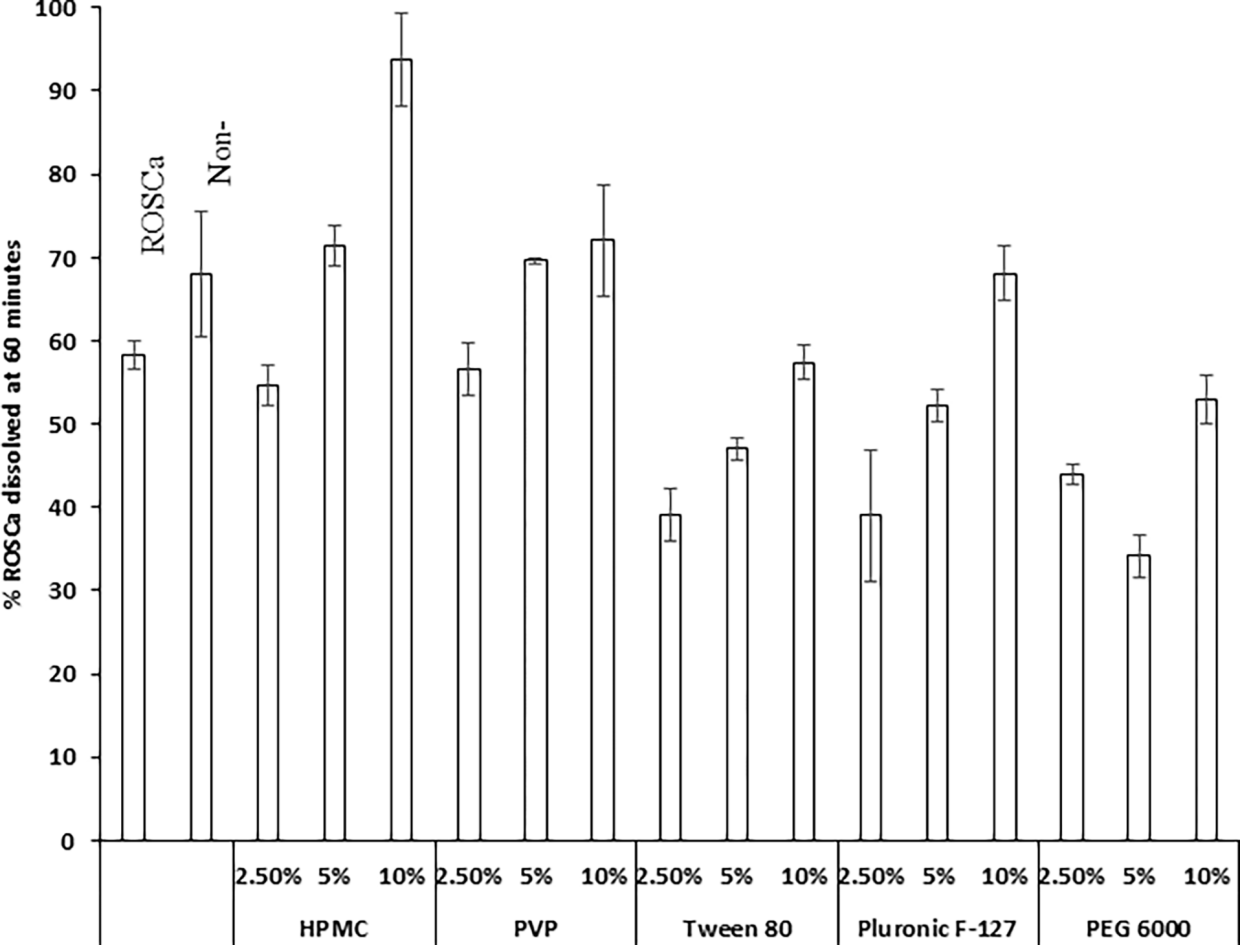
Effect of different stabilizers types and concentrations on the percent dissolved of ROSCa nanoparticles after 60 minutes compared to untreated ROSCa and non-stabilized ROSCa nanoparticles.

Fig 4 illustrates the percent of ROSCa dissolved after 60 minutes from nanoparticles stabilized with different stabilizers compared to both untreated ROSCa and the drug nanoparticles prepared without using stabilizer (0% stabilizer).

The dissolution profile of ROSCa in 0.1N HCl (pH 1.2) showed that only 58.25 ± 1.6% was dissolved after 1 hour. The initial dissolution rate after 5 min (IDR) was 24.45 ± 2.6% and a DE% after 60 minutes was 42.7. The dissolution rate of non-stabilized ROSCa nanoparticles was slightly higher than that of untreated ROSCa after 1h (%DE 52.1), (Table 1). Also, it was observed that at the low stabilizers' concentrations (2.5%), the drug dissolution from nanoparticles was not enhanced remarkably (except nanoparticle stabilized by 2.5% HPMC) compared to the untreated ROSCa and non-stabilized nanoparticles.

**Table 1:** Dissolution parameters of nanoparticles formulations stabilized with different concentrations of polymer compared to non-milled freeze-dried suspensions of ROSCa and the untreated drug.

ROSCa nanoparticles Formulation	IDR	RDR	DE% (60 min)	Non-milled Formulations	IDR	RDR	DE% (60min)	% ROSCa dissolved (60 min)
Untreated ROSCa	24.45	-	42.7					
Non-stabilized nano ROSCa	26.78	1.2	52.1					
HPMC (2.5%)	35.6	1.1	45.2	HPMC (2.5%)	37	1.2	52.6	65.23 ± 2.35
HPMC (5%)	39.7	1.4	58.8	HPMC (5%)	15.8	0.89	38.2	59.18 ± 5.53
HPMC (10%)	42.1	1.7	74.1	HPMC (10%)	29.4	1.2	51.3	63.99 ± 3.19
PVP (2.5%)	25.90	0.92	39.37	PVP (2.5%)	20.95	0.75	32.10	41.23 ± 0.39
PVP (5%)	38.76	1.25	53.56	PVP (5%)	22.19	1.00	42.93	57.61 ± 6.64
PVP (10%)	42.61	1.32	56.51	PVP (10%)	18.71	0.80	34.29	46.28 ± 3.11
Tween (2.5%)	21.92	0.66	28.31	Tween (2.5%)	15.28	0.44	19.20	26.42 ± 1.31
Tween (5%)	26.42	0.83	35.58	Tween (5%)	16.42	0.57	24.34	33.81 ± 2.08
Tweem (10%)	33.99	1.01	43.19	Tween (10%)	13.04	0.45	19.45	29.52 ± 7.93
Pluronic (2.5%)	22.04	0.69	29.49	Pluronic (2.5%)	22.19	0.69	29.66	37.19 ± 1.94
Pluronic (5%)	21.33	0.91	38.99	Pluronic (5%)	38.04	1.13	48.66	56.95 ± 3.28
Pluronic (10%)	27.99	1.18	50.70	Pluronic (10%)	82.95	1.76	75.34	80.04 ± 1.19
PEG (2.5%)	9.71	0.73	31.19	PEG (2.5%)	19.66	0.63	27.28	34.19 ± 2.57
PEG (5%)	16.71	0.57	24.43	PEG (5%)	18.95	0.71	30.50	40.28 ± 2.95
PEG (10%)	25.19	0.89	38.26	PEG (10%)	27.66	0.94	40.39	50.38 ± 3.86

The percent of ROSCa dissolved after 60 minutes from nanoparticles stabilized with high HPMC concentrations (5 and 10%) was higher than that of untreated ROSCa or non-stabilized ROSCa nanoparticles. Nanoparticles stabilized by 5% HPMC showed increased drug dissolution rate by 1.2-folds in comparison to that of the raw drug powder. Also, nanoparticles stabilized by 10% HPMC showed increased dissolution rate by 1.6-fold (P < 0.05). The solubilizing effect of HPMC on the drug with the reduced particle size may participate for enhancing the dissolution rate of ROSCa nanoparticles stabilized by the polymer. In contrast, the dissolution rate of ROSCa non-milled freeze-dried suspensions in HPMC solutions prepared without milling exhibited a slight improvement in comparison to the untreated drug. However, they still slower than that obtained with the corresponding nanoparticles prepared by milling, (Fig 4). The percent dissolved of ROSCa from non-milled freeze-dried ROSCa suspensions in 2.5% and 10% HPMC solutions were 65.23 ± 2.35% and 63.99 ± 3.19%, respectively.

The dissolution of ROSCa from nanoparticles stabilized by PVP showed a noticeable improvement in compared to that of the untreated drug, especially in case of using 5% and 10% PVP during nanoparticle formation. Nanoparticle formulations stabilized by 5% and 10% PVP significantly (P < 0.05) enhanced the dissolution rate of ROSCa in comparison with untreated raw drug powder. The values were 69.6 ± 3.3% and 72.08 ± 8.2% for 5% and 10%, respectively versus only 58.25 ± 1.62% for untreated ROSCa. In contrast, the dissolution of ROSCa non-milled freeze-dried suspensions in PVP solutions did not showed a significant enhancement of the drug dissolution compared to both untreated ROSCa and non-stabilized nanoparticle formula (Fig 4). Therefore, the enhancement of drug dissolution rate from nanoparticles stabilized by the same polymer can be attributed mainly due to the effect of nanosization. This happened by co-effects of decreasing the particle size by milling and the ability of PVP to stabilize the system through inhibiting both the aggregation and crystal formation [22]. The ability of PVP to reduce the crystallinity of ROSCa to more amorphous form, may participate in enhancing the dissolution rate. Rezaei and co-workers studied formulation of nanoparticles of indomethacin using PVP as polymeric stabilizer. The dissolution rate of indomethacin nanoparticles was higher compared to the micronized corresponding physical mixture. They attributed this finding to particle size reduction plus the transformation of indomethacin to amorphous form [27]. Kakran et al [10] showed that PVP inhibited the crystal growth and resulted in formation of amorphous drug particles.

The non-ionic surfactant Tween 80 was extensively utilized to enhance the dissolution rate of poorly soluble drugs by micelle formation. Unfortunately, the freeze-dried non-milled suspensions of ROSCa in different tween 80 concentrations did not show a pronounced enhancement of the dissolution rate of ROSCa (Fig 4). The percent of ROSCa dissolved from freeze-dried non-milled suspensions containing 2.5, 5 and 10% Tween were 26.42 ± 1.31%, 33.81 ± 2.08% and 29.52 ± 7.93%, respectively. This was compared to 58.25 ± 1.62% and 68 ± 7.6% for untreated ROSCa and non-stabilized ROSCa nanosuspension.

Tween 80 with the milling process produced nanoparticles with less than 500 nm in size. However, this marked decrease in the particle size did not greatly improve the drug dissolution rate compared to the untreated ROSCa (Fig 4). The lower zeta potential value of ROSCa nanosuspension stabilized by different tween concentrations, which might result in the aggregation of nanoparticles leading to formation of large size, might slow drug dissolution rate [30]. In addition, the drug dissolution rate increased with increasing the surfactant concentration, 39.1 ± 3.5%, 47 ± 0.32% and 57.4 ± 6.6% for nanoparticles stabilized by 2.5, 5 and 10% tween concentrations, respectively.

The milling of ROSCa in dispersions containing high concentrations (5% and 10%) of Pluronic F-127 succeeded to decrease the nanoparticles size to about 500 nm. However, this decrease in the particle size didn’t enhance the dissolution rate in case of nanoparticles stabilized by 5% pluronic F-127 solutions. In contrast, increasing the concentration of Pluronic F-127 to 10% resulted increasing the dissolution significantly from 39 ± 1.4% to 68 ± 3.8% in comparison to the untreated ROSCa dissolution (P < 0.05). The inefficiency of pluronic F-127 to enhance the dissolution rate of ROSCa in low concentrations could be attributed to nanoparticles agglomeration, which takes place after nanosization procedure resulting in slowing the drug dissolution rate [31].

The dissolution rate of ROSCa from freeze-dried non-milled suspensions containing different pluronic F-127 concentrations significantly (P < 0.05) increased by increasing the polymer concentration. The enhancement in the dissolution rate of ROSCa from non-milled freeze-dried suspension containing higher concentration of pluronic F-127 could be due to the wettability and solubilizing effect of the polymer. Since pluronic F-127 is non-ionic surfactant capable of forming miceller in the solution that enhanced drug solubility [32]. Thus, using higher concentration of pluronic F-127 alone could enhance the dissolution rate of ROSCa due to its wettability properties.

The dissolution profiles of nanoparticle formulations stabilized by different concentrations of PEG 6000 were slow (Fig 4). The results showed that increasing the concentration enhanced the percent dissolved of ROSCa after 2 hours. This enhancement was to 44 ± 2.8% for nanoparticles stabilized by 2.5% PEG 6000, 34.2 ± 1.5% for nanoparticles stabilized by 5% PEG 6000 and 53 ± 3.6% for nanoparticles stabilized by 10% PEG 6000. Also, PEG 6000 did not enhance the dissolution rate of ROSCa from non-milled freeze-dried suspension containing the polymer in comparison to the untreated form. The inefficiency of PEG 6000 to enhance the dissolution rate of ROSCa milled particles could be due large particle size produced by milling procedure, which in turns affected the dissolution rate in a negative way.

**Fig 5:**
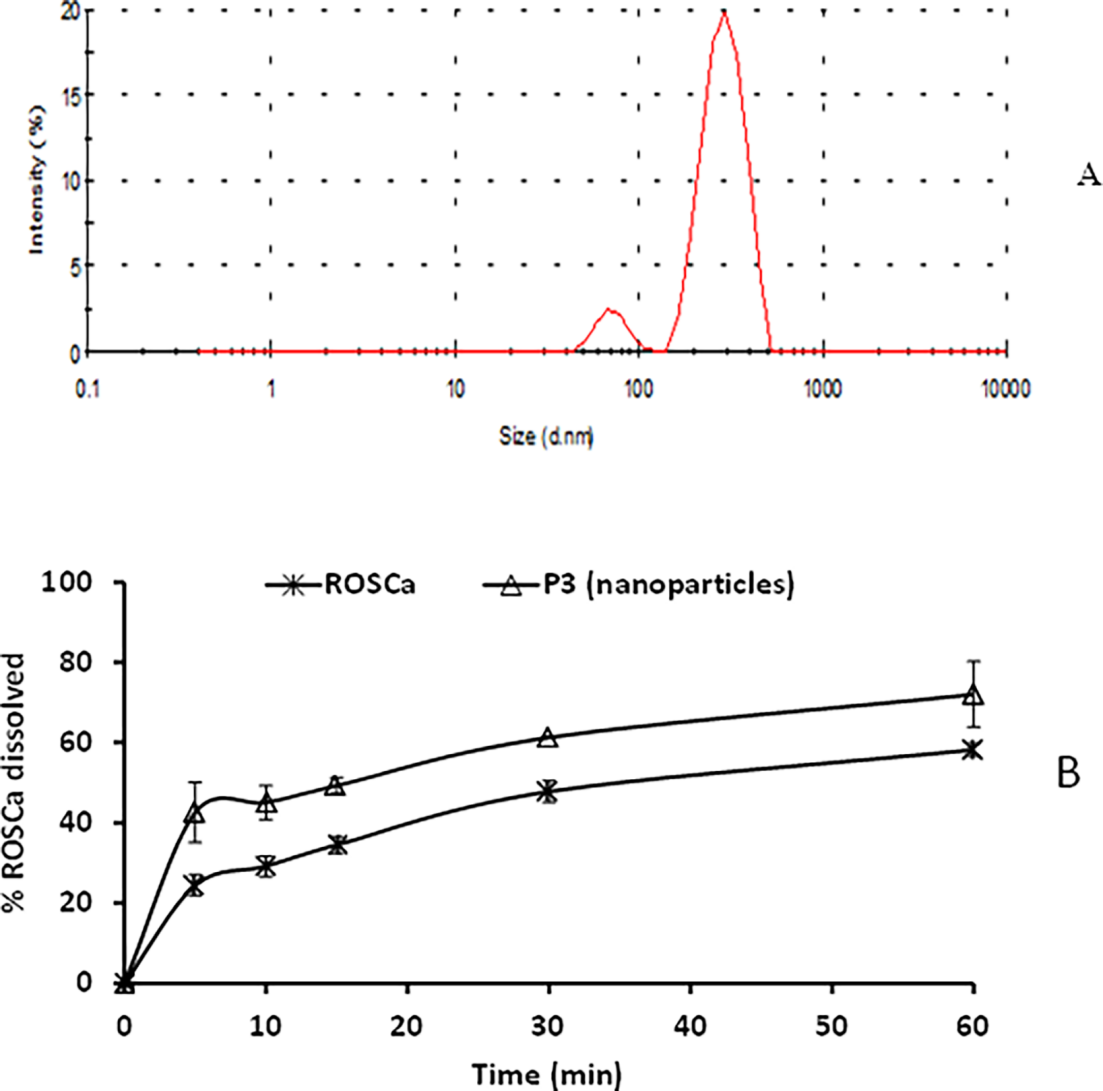
(A) Particle size of ROSCa nanoparticles prepared by planetary ball milling and stabilized by 10% PVP; (B) In vitro dissolution profile of ROSCa from nanoparticle formulation stabilized by 10% PVP, compared to the untreated drug in 0.1 N HCl at 37C°.

Based on the results of the particle size and the dissolution rate, it was found that nanoparticle formula stabilized by 10% PVP showed small particle size 461.8 ± 16.68 nm (Fig 5A), along with higher dissolution rate (72.04 ± 8.3% of ROSCa dissolved after 60 minutes) (Fig 5B). This was compared to 618 μm of untreated ROSCa with a percent dissolved of 58.25 ± 1.62% after 60 minutes. Therefore, this nanoparticle formula was subjected for XRPD and pharmacokinetic investigations.

### X-ray powder diffraction (XRPD)

In order to investigate the crystalline nature of the raw material, physical mixture and nanoparticles XRD studies were carried out. The XRPD pattern of untreated ROSCa showed two distinctive diffraction peaks at 2θ degree of 31.8 and 45.5 degrees. These sharp peaks indicate the lower crystallinity form of ROSCa.

**Fig 6:**
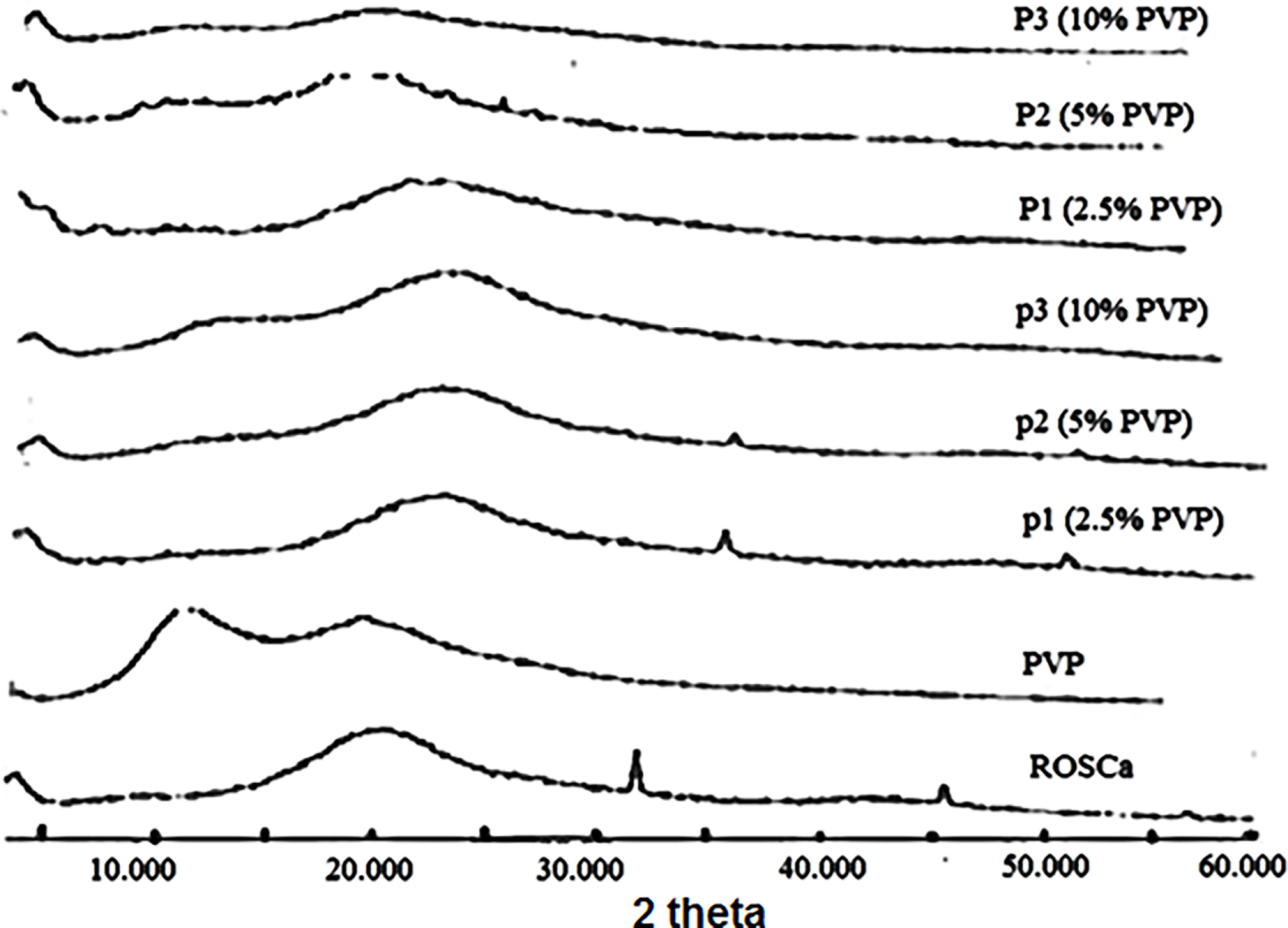
XRPD spectrum for ROSCa nanoparticles stabilized by different PVP concentrations compared to the corresponding non-milled freeze-dried suspensions and untreated ROSCa.

The XRPD spectra of non-milled freeze-dried suspensions in 2.5% and 5% solutions of PVP showed the existence of ROSCa characteristics peaks. Differently, these peaks disappeared in case of 10% PVP solution suggesting that increasing the PVP concentration transform ROSCa to amorphous form even in absence of milling. The addition of PVP as stabilizer for nanoparticles has a rule in converting the crystal form of ROSCa to amorphous one (Fig 6). In addition to its stabilizing effect, PVP was also used as colloidal stabilizer assisting in converting the drug substance from crystalline to amorphous forms.

### Pharmacokinetic studies

**Fig 7:**
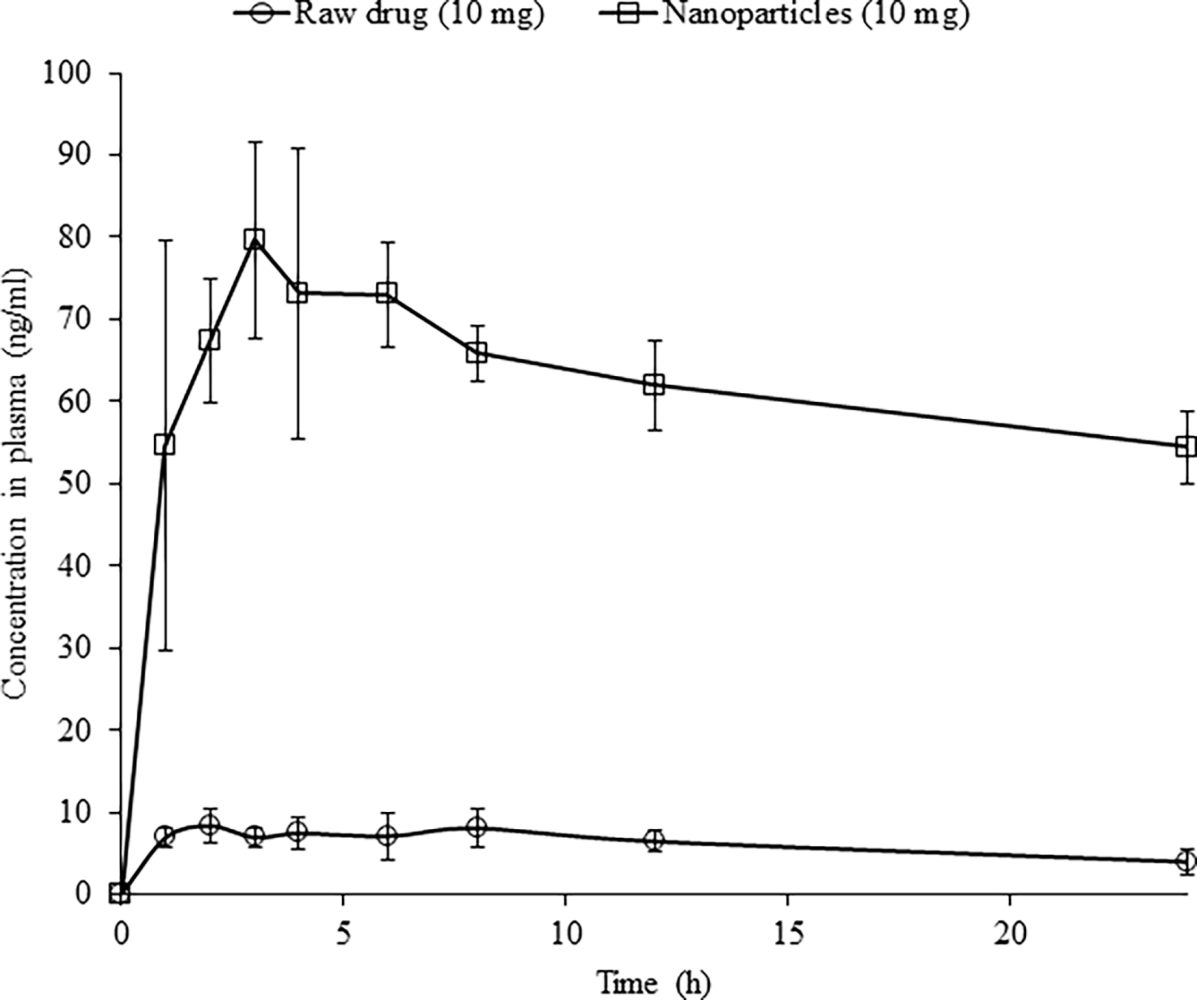
Plasma concentration-time curve of ROSCa in rabbits after oral administration of nanoparticle formula stabilized by 10 PVP compared to the raw drug.

The plasma concentration-time curve in rabbits after oral suspension administration of the prepared nanoparticle formula stabilized by 10% PVP was compared to raw drug (Fig 7). The ROSCa plasma concentrations for the prepared nanoparticle formula were significantly (*P* < 0.05) higher than those observed in case of raw ROSCa.

Pharmacokinetic parameters calculated from plasma concentration versus time profile of ROSCa are shown in Table 2. The maximum plasma concentration (C_max_) increased significantly by 8.2 folds after administration of nanoparticle formula (*P* < 0.05), in which C_max_ was 82.35 ng/ml compared to 9.2 ng/ml for the raw drug. The AUC_0-∞_ also increased by 21folds after administration of nanoparticles formulation (*P* < 0.05). The mean residence times (MRT) for nanoparticle formula and raw ROSCa were 80.24 and 30.11, respectively. The higher MRT may indicate that the drug may stay for long time inside the body as indicated by long half-life (55.06 ± 15.36 h) after nanoparticles administration. Moreover, lower elimination rate constant (0.013 ± 0.004) was observed in case of nanoparticle formulation compared to that observed for raw drug (0.04 ± 0.033).

**Table 2:** Pharmacokinetic parameters of ROSCa in rabbits after oral administration of nanoparticle formula stabilized by 10% PVP compared to the raw drug.

Parameters	Raw ROSCa	ROSCa Nanoparticle formula	*P*-value
T_max_ (h)	3.7 ± 3.09	4 ± 1.4	0.44
C_max_ (ng/ml)	9.2 ± 2.04	82.35 ± 13.27	0.0000
AUC_0-t_ (ng/ml*h)	148.06 ± 29.3	1478.09 ± 80.65	0.000
AUC _0-inf_obs_ (ng/ml*h)	276.6 ± 108.5	5854.25 ± 1381.18	0.0001
MRT (h)	30.11 ± 12.44	80.24 ± 22.86	0.0042
T_1/2_ (h)	19.94 ± 9.2	55.06 ± 15.36	0.0039
K_el_ (h^-1^)	0.04 ± 0.033	0.013 ± 0.004	0.0514

## Conclusion

ROSCa nanoparticles were successfully formulated by wet milling technique using planetary ball mill. The type and concentration of stabilizers used in nanoparticle formulation could be monitored to attain the desired nanoparticles attributes such as particle size, zeta potential as well as dissolution parameters. The pharmacokinetic data showed that both C_max_ and AUC increased significantly by 8.2-folds and 21.1-folds in case of ROSCa nanoparticles stabilized by 10% PVP compared to the untreated ROSCa (P<0.05). Therefore, formulation of ROSCa stabilized nanoparticles resulted in enhancing the drug dissolution and improved its oral bioavailability.
